# The functional architecture of mother-infant communication, and the development of infant social expressiveness in the first two months

**DOI:** 10.1038/srep39019

**Published:** 2016-12-14

**Authors:** Lynne Murray, Leonardo De Pascalis, Laura Bozicevic, Laura Hawkins, Valentina Sclafani, Pier Francesco Ferrari

**Affiliations:** 1 University of Reading, School of Psychology & Clinical Language Sciences, Reading, RG6 7BE, UK; 2 Stellenbosch University, Department of Psychology, Stellenbosch, 7600, South Africa; 3 University of Cape Town, Department of Psychology, Cape Town, 7701, South Africa; 4 University of Liverpool, Institute of Psychology Health and Society, Liverpool, L69 7ZA, UK; 5 Università di Parma, Dipartimento di Neuroscienze, Parma, 43125, Italy; 6 Institut des Sciences Cognitives Marc Jeannerod, CNRS, Bron, France

## Abstract

By two-three months, infants show active social expressions during face-to-face interactions. These interactions are important, as they provide the foundation for later emotional regulation and cognition, but little is known about how infant social expressiveness develops. We considered two different accounts. One emphasizes the *contingency* of parental responsiveness, regardless of its form; the other, the *functional architecture* account, emphasizes the preparedness of both infants and parents to respond in specific ways to particular forms of behaviour in their partner. We videotaped mother-infant interactions from one to nine weeks, and analysed them with a micro-analytic coding scheme. Infant social expressiveness increased through the nine-week period, particularly after 3 weeks. This development was unrelated to the extent of maternal contingent responsiveness, even to infant social expressions. By contrast, specific forms of response that mothers used preferentially for infant social expressions-mirroring, marking with a smile- predicted the increase in these infant behaviours over time. These results support a functional architecture account of the perceptual and behavioural predispositions of infants and parents that allow young infants to capitalize on relatively limited exposure to specific parental behaviours, in order to develop important social capacities.

Infants’ capacity to engage in social interactions is fundamental to their psychological development, and includes the perception and appropriate expression of social cues. Regarding perception, even newborns prefer stimuli signaling readiness to interact (e.g., direct gaze, happy expression, infant–directed speech)^1^,^2^,^3^, preferences that develop in response to the environment^4^,^5^,^6^. Yet, little is known about the early development of social *expressiveness* since, with few exceptions^7^, studies have generally started only after two-three months postpartum. By this age, however, infants already show complex social behaviours during interactions (e.g., smiles, positive vocalizations, active mouthing termed ‘prespeech’)^7^,^8^. These interactions are important, as they provide the foundations for emotion regulation and cognition, and already show effects of parental and infant clinical conditions^9^,^10^ that predict long-term functioning^11^. Understanding their development through the postnatal weeks is, therefore, critical, since this may reveal markers of clinical difficulties, which in turn could open up avenues for early intervention. In particular, it is important to establish the extent to which the infant’s capacity for engagement at two-three months arises spontaneously, without continuity with previous social experience, or whether certain parental behaviours are important for its emergence.

Our study aimed to address this question. We charted the evolution of infant social expressiveness through the first two months, and investigated whether parental behaviour contributed to its development. In doing so, we considered two different perspectives. The first draws on a long tradition in the history of child development^12^ that emphasizes the *contingency,* rather than the form, of parental responsiveness as shaping the infant’s behaviour, including their social expressiveness. This perspective is illustrated in the seminal study of Watson^13^,^14^, showing that infant behaviours considered prototypically social (smiling, cooing etc.) increased in frequency even with *non-social* contingent feedback, and has remained a prominent theme in recent accounts of parental responsiveness and infant social development^15^,^16^,^17^. The second, *functional architecture,* account while recognizing the role of parental contingency, emphasizes the experience-expectant nature of early social engagement. In this view, infants are not only predisposed to *attend* to particular social stimuli^5^, but also to respond in certain ways to specific forms of parental display, as part of a general intersubjective capacity^8^,^18^,^19^,^20^,^21^,^22^. By corollary, this account notes the preparedness of parents to respond to infant cues with specific behaviours to which infants, in turn, are particularly sensitive. These parental responses include ‘mirroring’, and ‘marking’ (behaviours highlighting infant actions with attention-attracting cues like eyebrow flashes)^23^. Thus, a mutual ‘social fittedness’^24^ is proposed, in which the perceptual propensities and behavioural repertoires of infants and parents together provide a functional architecture that supports infant social development.

To evaluate these two accounts, we video-recorded mother-infant face-to-face interactions from 1–9 weeks, and coded a comprehensive set of infant facial and vocal behaviours [positive social expressions (smiles, vocalizations and pre-speech mouth movements), biological events (sneezes, hiccoughs etc.), expressions of negative affect, and non-social mouth movements]. For each one, we recorded the presence/absence of a contingent maternal response, and its form [i.e., mirroring, marking (both positive- i.e., with a smile, and neutral- i.e., without a smile), and negative responses] (see Supplementary Methods for details of the coding scheme). Using these data, we charted the development of infant social expressiveness, and we investigated the contingency of maternal responses, and their prominence relative to non-contingent maternal behaviour, and analysed their occurrence according to infant age and behaviour. We conducted similar investigation of the different forms of maternal response, again looking at the influence of infant age and behaviour, and we also estimated the specificity of associations between different maternal and infant behaviours. Finally, we examined the influence of both the contingency and the form of maternal responsiveness on infant social expressiveness, both within the same interaction, and over time.

## Results

### Change in infant social expressiveness over the first 9 weeks

**Figure 1:**
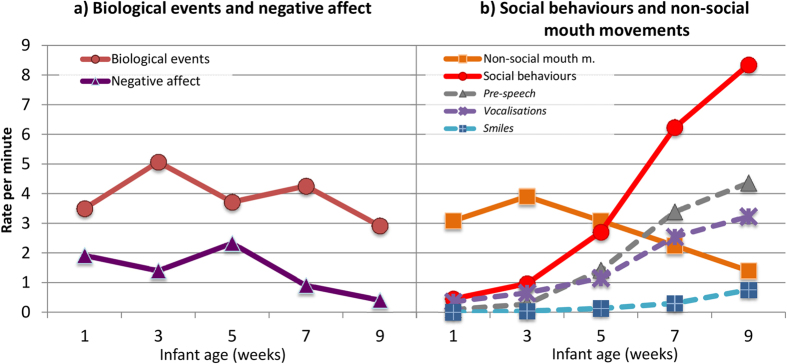
Change during the first 2 months of life in infant (**a**) biological events, and negative affect, and (**b**) non-social mouth movements and social behaviours (component behaviours of this category are also shown, as dashed lines).

Figure 1 (see Supplementary Table S1 for details) shows a profile, over time, of the rate of occurrence per minute of the different infant behaviours (confirmed by PCA, Supplementary Results). Biological behaviours showed no systematic effect of age, with change, albeit significant [Age: *Χ*^*2*^(4) = 21.575, *p* < 0.001, *R*^*2*^ = 0.050], being erratic between consecutive observations (i.e. increasing from 1 to 3 weeks: *p* = 0.012, and decreasing from each of 3 and 7 weeks to 9 weeks *p* = 0.001, and *p* = 0.011, respectively). Similarly, expressions of negative affect, although decreasing overall with time [Age: *Χ*^*2*^ (4) = 103.062, *p* < 0.001, *R*^*2*^ = 0.225; 1 vs. 9 weeks: *p* < 0.001], did not change consistently between observations. Infant social expressiveness, and non-social mouth movements, by contrast, changed clearly with time. The former increased over the first nine weeks [Age: *Χ*^*2*^ (4) = 679.828, *p* < 0.001, *R*^*2*^ = 0.747; 1 vs. 9 weeks: *p* < 0.001], and this was true for each individual social behaviour-between 3 and 9 weeks for pre-speech [Age: *Χ*^*2*^ (4) = 429.330, *p* < 0.001, *R*^*2*^ = 0.720; 3 vs. 9 weeks: *p* < 0.001], between 5 and 9 weeks for smiles [Age: *Χ*^*2*^ (4) = 78.660, *p* < 0.001, *R*^*2*^ = 0.973; 5 vs. 9 weeks: *p* < 0.001], and over the whole 9-week period for vocalizations [Age: *Χ*^*2*^ (4) = 207.410, *p* < 0.001, *R*^*2*^ = 0.441; 1 vs. 9 weeks: *p* < 0.001]. Unlike social expressiveness, infant non-social mouth movements decreased between weeks 3 and 9 [Age: *Χ*^*2*^ (4) = 72.228, *p* < 0.001, *R*^*2*^ = 0.194; 3 vs. 9 weeks: *p* < 0.001].

### The extent of prominence and contingency of maternal responses to the infant

**Figure 2:**
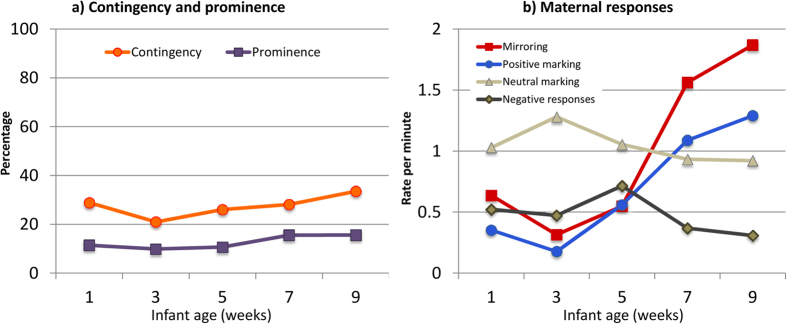
Change during the first 2 months in (**a**) the contingency (percentage of infant behaviours responded to by the mother), and prominence (percentage of maternal behaviours that comprise responses to the infant) of maternal responsiveness, and (**b**) the rate of the different kinds of maternal responses to infant behaviour.

We then considered maternal behaviour, first determining its prominence and contingency. For prominence, we examined maternal behaviours that were responses to the infant as a percentage of all maternal behaviours coded (i.e., responses plus non-responsive behaviours). There was no effect of infant age (*p* = 0.070); the percentages for each time point ranged from 9 to 15% (see Fig. 2a, and Supplementary Table S2 for details), indicating that responses to the infant represented only a small proportion of the total corpus of maternal behaviour. For contingency, we examined the percentage of infant behaviours that were responded to by the mother at each time point, and this ranged from 20 to 33% (see Fig. 2a, and Supplementary Table S2 for details), suggesting that the infant’s overall experience of contingent responsiveness, while gradually increasing over time [Age: *Χ*^*2*^ (4) = 13.240, *p* = 0.010, *R*^*2*^ = 0.106; 3 vs. 9 weeks: *p* = 0.004], was never particularly high.

### Rates of maternal contingent responsiveness to different infant behaviours

Although rather few infant behaviours in general (27.62%), received a maternal response, it seemed likely that mothers would respond differentially to different infant behaviours. We investigated the likelihood of the mother responding as a function of the type of infant behaviour, the infant’s age, and the behaviour x age interaction term. Only the main effect of infant behaviour was significant [*Χ*^*2*^ (3) = 222.582, *p* < 0.001, *R*^*2*^ = 0.156]: mothers responded to infant social behaviour and expressions of negative affect to a similar degree (36.93% and 38.11%, respectively), and to a greater extent than to biological events (27.73%) (both *p’s* < 0.001); and they responded to all these infant behaviours more than to non-social mouth movements, which received few responses (7.70%) (all *p’s* < 0.001).

### Changes in the form of maternal responses over time

As well as the *extent* of maternal responsiveness, we investigated its form. Figure 2b shows the occurrence over time of the different categories of maternal response (these were broadly confirmed by PCA, although mirroring and positive marking, which we distinguished on theoretical grounds, loaded on the same component, see Supplementary Results). Mirroring and positive marking increased with infant age - the former from 3 to 9 weeks [Age: *Χ*^*2*^ (4) = 109.655, *p* < 0.001, *R*^*2*^ = 0.263; 3 vs.9 weeks: *p* < 0.001], and the latter over the whole period [Age: *Χ*^*2*^ (4) = 92.174, *p* < 0.001, *R*^*2*^ = 0.390; 1 vs. 9 weeks *p* < 0.001]. Neutral markings, by contrast, showed no effect of time. Negative responses were infrequent, and apart from a small spike at 5 weeks, they decreased over time [Age: *Χ*^*2*^ (4) = 9.645, *p* = 0.047, *R*^*2*^ = 0.043; 5 vs. 9 weeks: *p* < 0.032].

### The extent and nature of specificity in associations between infant behaviours and maternal responses

We next considered the relationships between the different maternal and infant behaviours, in two sets of analyses. First, we examined how mothers distributed their responses, according to the kind of infant behavior (also testing for effects of infant age, and controlling for the base rate of infant behaviours). There were no main or interactive effects of age.

**Figure 3:**
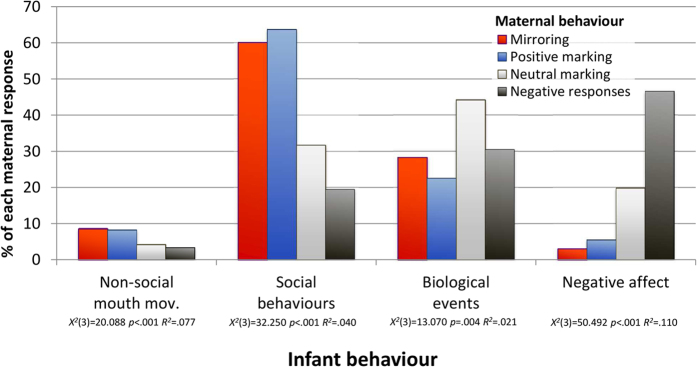
Distribution of each kind of maternal response (absolute percentages) according to infant behaviour (statistics, and p-value for the difference among maternal behaviours are reported for each infant behavior).

Figure 3 shows that, for all four infant behaviours, mothers used their different responses to different degrees (see Supplementary Table S3 for details). Thus, when the infant showed social behaviour, mothers deployed a far higher percentage of their mirroring and positive marking responses (60.07% and 63.74%, respectively) than their neutral marking (31.68%) and negative (19.49%) responses (for all these pairwise comparisons, *p* ≤ 0.008). Similar specificity of response was shown for infant negative affect, where mothers used a greater percentage of their negative (46.61%) vs. other responses (each < 20%; pairwise comparison *p*’s ≤ 0.015). With regard to infant biological events, mothers again used their different responses unevenly, although, in this case, relationships were somewhat less clear-cut: thus, while they used a higher percentage of their neutral (44.27%) vs. their positive markings (22.53%; *p* = 0.004) and, albeit marginally, mirroring responses (28.36%; *p* = 0.059), they used a similar percentage of their negative responses (30.51%; *p* = 0.332). Notably, mothers never deployed more than 9% of any response to infant non-social mouth movements, although when they did respond, the pattern was again uneven, and similar to that for infant social behaviour (i.e., mirroring and positive marking occurred more commonly than both neutral marking and negative responses, pairwise comparisons *p*’s all ≤0.050).

**Figure 4:**
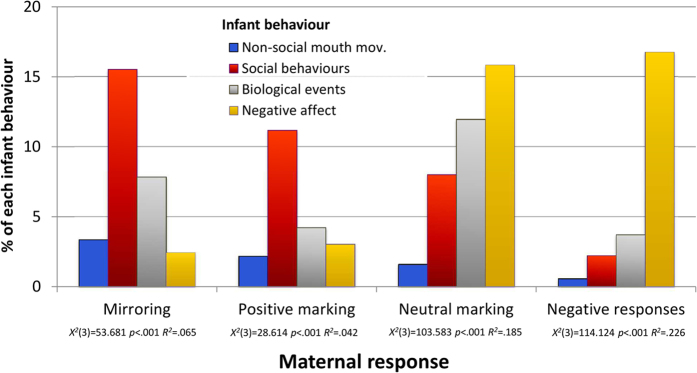
Absolute percentages of infant behaviours responded to with each kind of maternal response (statistics, and p-value for the difference among infant behaviours are reported for each maternal behavior).

In our second set of analyses, we examined whether the infant experienced each kind of maternal response differently, according to which behaviour the infant had performed. Thus, for each kind of maternal response, we compared the different infant behaviours according to how frequently the maternal behaviour in question followed. There were no main or interactive effects of age. Figure 4 shows that, for all maternal behaviours, response rates differed significantly across the four infant behaviours (see Supplementary Table S4 for details). Most maternal responses (three of four) occurred principally following just one kind of infant behaviour. Thus, maternal mirroring was more likely to follow infant social expressions than any other infant behaviour (i.e., after 15.53% vs. after <8% the others; all pairwise comparison *p*’s < 0.001), and the same pattern obtained for maternal positive marking (i.e., it occurred after 11.19% infant social expressions vs. after <5% other behaviours; pairwise comparisons were all *p* ≤ 0.002, apart from that with infant negative affect, which was infrequent, where *p* = 0.19). We found a similarly specific association for maternal negative responses, which were more likely to occur following infant negative affect than after any other infant behaviour (i.e., 16.77% vs. <4%; all pairwise comparison *p*’s < 0.001). Only maternal neutral marking lacked a clear association with a particular infant behaviour: thus, although this response most often followed infant negative affect (15.85%), and did so more than after non-social mouth movements (1.60%; *p* < 0.001) or, albeit marginally, infant social expressions (8.00%; *p* = 0.057), it occurred after biological events to a similar degree (11.96%; *p* = 0.296).

### Effects of maternal responses on infant social behaviours

Finally, we examined the effects of maternal behaviour on infant social expressiveness. We first looked at *immediate effects*, and, second, at *effects over time*–i.e., from one assessment to the next. At weeks 1 and 3, the rates of both infant social expressiveness and certain forms of maternal response (i.e., mirroring and positive marking) were particularly low (see Figs 1 and 2). Accordingly, to ensure enough variability in both the dependent variable and predictors, we restricted analyses to weeks 5 to 9.

#### Immediate effects

We investigated the immediate effect of any contingent response compared to no response, and also the effect of different forms of maternal response, by examining the length of time (in seconds) between consecutive instances of infant social behaviour, according to the mother’s behaviour following the first instance. Testing effects of any *vs*. no maternal response, infant age and their interaction, we found only main effects. For age [*Χ*^*2*^ (2) = 14.613, *p* < 0.001, *R*^*2*^ = 0.135], the older the infant, the shorter the time between consecutive social behaviours [Week 5 M(SD) = 12.95(14.55) vs. Week 9 M(SD) = 6.65(7.67); *p* < 0.001]. For maternal response [*Χ*^*2*^ (1) = 7.919, *p* = 0.005, *R*^*2*^ = 0.017], shorter intervals occurred between consecutive infant social behaviours when the mother responded to, rather than ignored, the first instance [Ignoring M(SD) = 8.81(11.98) vs. Responding M(SD) = 6.86(9.41); *p* = 0.005]. We then investigated whether this effect of contingent responsiveness was true for *all* forms of maternal response, distinguishing mirroring, positive and neutral marking, negative responses, and ignoring (i.e. no response). Again, there were no interaction effects, and the main effect of age remained significant [*Χ*^*2*^ (2) = 13.713, *p* = 0.001, *R*^*2*^ = 0.129]. The main effect of the form of maternal response was also significant [*Χ*^*2*^ (4) = 11.297, *p* = 0.023, *R*^*2*^ = 0.020], with only positive marking bringing about shorter times between infant social behaviours than ignoring them [Ignoring M(SD) = 8.81(11.98) vs. Positive Marking M(SD) = 6.45(10.85); *p* = 0.043; all other kinds of maternal response vs. Ignoring, *p*’s > 0.114]. To ascertain the relevance of contingency, independently of the form of responsiveness, we re-ran the first model, including main effects of age and maternal response, and a variable isolating the effect of positive marking: this rendered the previous main effect of maternal contingent responsiveness non-significant.

#### Effects over time

**Figure 5:**
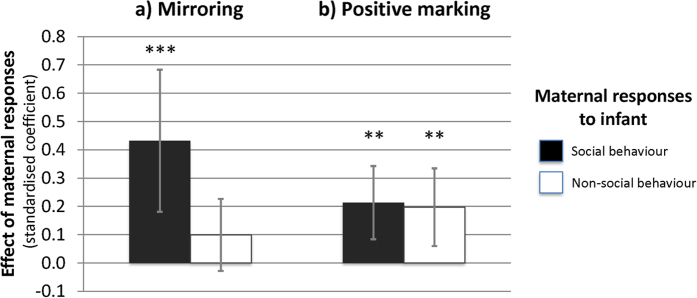
Standardized coefficients for the effects of maternal responses at time *t* on infant social behaviour at time *t* +* 1*, when mothers use (**a**) mirroring, (**b**) positive marking. (**p ≤ :010; ***p ≤ :001).

To determine the role of maternal responsiveness in the development of infant social behaviour over time, we examined the effect of responses at time *t* on the rate of infant social behaviours at time *t + 1* (i.e., at the next assessment point), controlling for infant social behaviours at time *t* (see Supplementary Table S5). We first examined the effects of the overall level of contingent responsiveness (i.e., the percentage of infant behaviours the mother responded to), regardless of its form. No effect on the development of infant social behaviour was found. Nevertheless, since maternal contingent responsiveness to infant *social* behaviours might be expected to be more influential than their general responsiveness, we ran two further models, distinguishing effects of responsiveness to social *vs.* non-social behaviours, but neither was significant (*p* = 0.611 and *p* = 0.117, respectively). Using similar models, we then tested the effects on the development of infant social expressiveness of specific maternal responses, again distinguishing those to infant social *vs.* non-social behaviour. More maternal mirroring responses to infant social, but not non-social, behaviour, predicted higher rates of social behaviour at the subsequent assessment [*Χ*^*2*^ (1) = 11.598, *p* < 0.001, *R*^*2*^ = 0.120] (see Fig. 5). Maternal positive marking was similarly associated with a higher rate of infant social behaviour two weeks later. In this case, the increase in infant social expressiveness occurred, albeit with standardized coefficients that were half the size of that for maternal mirroring, regardless of whether mothers used positive marking to respond to infant social, or non-social, behavior ([*Χ*^*2*^ (1) = 10.725, *p* = 0.001, *R*^*2*^ = 0.069] and [*Χ*^*2*^ (1) = 7.993, *p* = 0.005, *R*^*2*^ = 0.051], respectively) (see Fig. 5). In contrast to these positive effects of mirroring and positive marking (and bearing in mind the general increase in infant social behaviours over the first 9 weeks (Fig. 1)), more maternal neutral marking, whether to infant social or non-social behaviour, was associated with a relative reduction in the rates of infant subsequent social expressiveness ([*Χ*^*2*^ (1) = 6.667, *p* = 0.010, *R*^*2*^ = 0.053], and [*Χ*^*2*^ (1) = 8.705, *p* = 0.003, *R*^*2*^ = 0.050], respectively). Finally, more frequent use of maternal negative responses was also associated with a relative reduction in infant social expressiveness at the next assessment, regardless of whether they occurred in response to social [*Χ*^*2*^ (1) = 6.987, *p* = 0.008, *R*^*2*^ = 0.074], or non-social infant behaviour [*Χ*^*2*^ (1) = 14.538, *p* < 0.001, *R*^*2*^ = 0.075].

## Discussion

Our study of naturalistic mother-infant interactions through the first nine weeks showed infant social expressiveness to be highly structured, combining different individual behaviours (i.e. vocalizations, smiles and prespeech mouth movements) in a single group, distinct from other mouth movements. Further, consistent with a previous descriptive study^7^, this social expressiveness progressively increased, particularly after three weeks. Our key focus was to investigate the role of maternal responsiveness, using a micro-analytic coding scheme that distinguished different maternal behaviours highlighted in the literature, and that allowed us to evaluate two different accounts of social development.

The first account emphasizes the frequency of parental contingent responsiveness during early interactions, and its potential to reinforce the occurrence of infant behaviours, including their social expressions^15^,^16^. We found no evidence to support this account. In fact, only a small proportion of maternal actions during engagements with their infant was responsive to infant behaviour, i.e., responsive behaviours were not highly prominent; and, in turn, only a minority of infant behaviours, including social expressions, met with a contingent maternal response. This is particularly striking when one considers that mothers were specifically invited to engage socially with their infant during our observations. Critically, moreover, once the form of maternal response was taken into account, the contingency of maternal behaviour was unrelated to the infant’s repetition of their social expressions within the same interaction; further, levels of maternal contingency, even when considered specifically in relation to infant social behaviours, were unrelated to future infant social development.

In contrast to the null findings for overall rates of contingent maternal responsiveness, we found its *form* to be important. First, mothers systematically deployed specific responses to different infant behaviours: thus, they used mirroring and positive marking (i.e., marking accompanied by smiling) to respond to infant social behaviours, whereas neutral marking (i.e., without smiling) was used principally for biological events; and negative responses were used mainly in relation to expressions of infant negative affect. In turn, the maternal responses infants received systematically varied according to the infants’ own actions. Second, and of particular importance, those behaviours that mothers used preferentially to respond to infant social expressions, that is, marking with a smile and mirroring, were also the behaviours that increased the occurrence of these infant expressions, the former in both the immediate and the long term, the latter in the long term, and with greater effect.

Together, these findings provide evidence for an early functional architecture for parent-infant communication, underpinned by the close fittedness between the two participants’ perceptual and behavioural propensities. Further, they underline the infant’s preparedness to capitalize on specific, relatively infrequent, forms of maternal response in the process of developing their social expressiveness. Thus, our findings complement the experimental evidence on the preferential responsiveness of newborn infants to cues signaling readiness for social engagement^5^ and, correspondingly, the specialized sensitivity of adults to infant characteristics^23^. They are also consistent with experiments with human infant, and non-human adult primates, showing a preference for imitative vs. non-imitative forms of contingent response^20^,^21^,^25^,^26^. The neuronal and physiological mechanisms involved in these mother-infant interactive responses, including the vagal regulation of infant autonomic state, are being increasingly established^27^,^28^,^29^,^30^. What the present study adds is a detailed elucidation of the *behavioural* processes whereby these basic infant and parent perceptual propensities become harnessed to support the development of infant social expressiveness in the first weeks of life.

To understand this functional architecture for communication more fully, we need to consider further the components of maternal responsiveness. With regard to mirroring, given that this behaviour increases the engagement and affiliation of recipients, including infant primates^31^,^32^,^33^, the question arises whether its positive effect on the development of infant social expressiveness in our study was of this generic, motivational, kind. This seems unlikely, because only maternal mirroring of social, but not non-social, behaviours increased subsequent infant social expressiveness. Rather, the specificity of this association suggests that maternal mirroring enhanced infant control over the production of mirrored social expressions. This effect may involve neural mechanisms, present in both adult and newborn primates, that map observed and executed facial gestures^6^,^28^,^34^,^35^. Indeed, the preparedness of infants to benefit from maternal mirroring is underscored by the fact that only 15% of their social expressions received this response, yet such mirroring had a notable impact on infant social behaviour two weeks later, even in the context of the significant endogenous developmental push towards increasing social expressiveness.

Turning to maternal positive marking, this behaviour was strongly related to mirroring, and predominantly used in the same context (i.e., to infant social expressions), but its effects were somewhat different. Thus, it elicited rapid repetition of infant social behaviour within the same interaction, as well as increasing its expression over time. Notably, this long-term effect occurred if mothers showed this response to *non-social* infant behaviours, as well as their social expressions. Smiles, which defined this category of marking, comprise potent stimuli, being discriminated and preferred over neutral expressions even by neonates^2^; and while they are associated with positive affect in general, there is also evidence for their specifically *social* function^36^,^37^. It seems likely in our study that the combination of this intrinsic social reward with the attention-attracting cues entailed in marking, reinforced the infants’ motivation to become more socially engaged themselves. Further, since infants develop expectations for particular interactive experiences^38^, if mothers show this behaviour, infants may subsequently expect further rewarding experiences, and increase their social expressions accordingly.

Notably, the literature on adult marking behaviour does not always distinguish between instances that are, and are not, accompanied by smiles^9^,^39^. Our study showed this distinction is important: thus, the two kinds of marking were unrelated in their occurrence, used in different interactive contexts, and had different effects on infant social expressiveness. The fact that marking without smiles occurred principally in response to infant biological events (e.g., ‘*What* a big sneeze’), suggests that it might be a precursor of the ostensive marking cues employed with older infants to facilitate their shift in attention to a common focus^40^. That is, its function may be essentially *referential*, rather than *relational*. Before two months, infants may have only limited abilities to capitalize on the more complex, referential, uses of this parental behaviour, and this may explain why its deployment failed to enhance infant engagement.

Maternal negative responses were infrequent, unrelated to other maternal behaviours, occurred mainly following expressions of infant negative affect, and had a negative impact on infant social development. This might be because infants find certain negative responses directly aversive (e.g., hostile tone of voice, angry facial expression), reducing their motivation to engage. It is also possible that infants are predisposed to expect responses within a particular range (e.g., in terms of their intensity, valence and form), and that violation of such expectations^18^,^19^,^41^, even if not intrinsically aversive (e.g., exaggerated maternal laughter in response to infant pre-speech), may also disrupt infant social engagement. This suggestion is consistent with the negative impact on infant engagement of mis-attuned maternal responses in clinical populations^9^,^18^.

Determining the nature and limits of infant social expectations is important for understanding their intersubjective capacities. Possible avenues would be conducting experimental studies on infant responses to different social displays, and examining cross-cultural variations in parent-infant engagements and their effects on later social functioning. For example, in cultures where parents predominantly use touch vs. facial expressiveness during social interactions, infants develop different profiles of engagement from those in more visually responsive populations^42^. Establishing what differentiates adaptive variations in responsiveness, as between cultures, from those with adverse clinical consequences could be particularly illuminating.

Finally, the early communicative patterns we observed appear to have a long evolutionary history: recent research shows that some non-human primates exhibit complex mother-infant interactions (e.g., with lip-smacking, mutual gaze)^43^,^44^,^45^ that, as with humans, provide the basis for later socio-emotional functioning.

## Conclusions

Our results are consistent with a functional architecture for mother-infant engagements that underlies infant social development. Thus, the preparedness of the infant’s perceptual system to attend to particular social stimuli is matched by the propensity of parents to deploy specific responses to different infant behaviours that typically embody their characteristic ‘vitality dynamics’^46^. Further, infants appear to capitalize on a limited *corpus* of these responses to develop their social expressiveness. In contrast, our findings were at variance with an account of the development of infant social expressiveness in terms of the contingency of parental behaviour alone, since contingent responses, including those to infant social expressions, did not predict later infant social behaviour. This is not to say that contingent associations are irrelevant - parental responses that fostered the development of social expressiveness were, by definition, contingent. Rather, our data show that the *form* of parental response is critical, indicating the experience-expectant nature of the development process. This conclusion is underlined by the similarities between our observations and recent evidence on non-human primate mother-infant interactions.

Finally, our findings have potentially important clinical implications. Parent-infant interactions in clinical contexts (e.g., depressed parents, those with early traumatic experiences, or infants with clinical conditions such as cleft lip) show significant difficulties at two-three months that predict later poor child functioning^9^,^10^,^47^. The current study shows that these interactions are the outcome of a process starting in the first weeks. It might, therefore, be profitable to mobilize early identification of at-risk populations, to support parent-infant contacts that foster infant positive social engagement and future good outcome.

## Methods

More details are provided in Supplementary Methods.

### Participants

Twenty healthy mother-infant dyads [12 male infants; mothers’ age: M(SD) = 33.70(2.73)] were recruited at the Royal Berkshire Hospital (UK), and gave written informed consent. The study was conducted according to the British Psychological Society’s Code of Human Research Ethics, and approved by the Ethics Committee of the University of Reading (n. 11/45).

### Procedure

**Figure 6:**
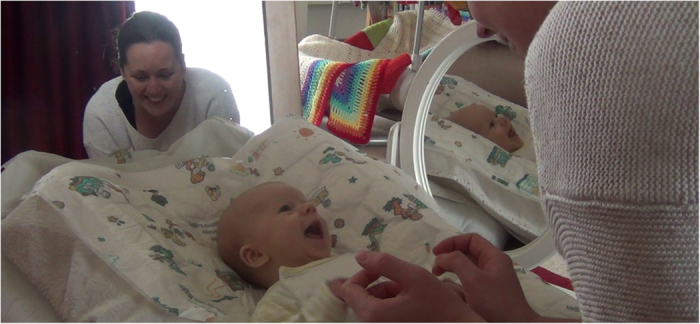
Mother-infant interaction setting.

In home visits at 1, 3, 5, 7 and 9 weeks postpartum, 3 minutes of mother-infant face-to-face interaction were video-recorded when infants were calm and alert. Infants were placed semi-reclined on a mat on the floor, and mothers sat opposite, leaning towards their infant. A camera filmed the infant’s face and upper body, and a reflection of the mother’s face and upper body in a mirror placed behind the infant; another mirror placed alongside the infant showed their face if they turned away (Fig. 6).

### Coding

Videos were event-coded on a one-second time base, using purpose-built software. Codes included key, mutually exclusive, infant and maternal events described in the literature on mother-infant interactions^8^,^9^,^19^. Infant events were clearly discernible, discrete behaviours with definite onset, thus readily identifiable by the mother in live time. Maternal contingent responses were coded as events occurring within two seconds of each infant event^9^,^39^. Maternal non-contingent, spontaneous, behaviours were also coded as events (e.g., vocalizations, smiles, tongue protrusions, mouth openings, head nods).

### Data analysis

We used two-level random intercept models to analyse the effect of infant age on infant and maternal behaviours (Poisson model, with a Log link, and interaction time as offset), and on the prominence and contingency of maternal responsiveness (Gaussian model, with an Identity link).

We used three-level random intercept Binomial models, with a Logit link (including main and interactive effects of infant age) to compare i) the likelihood of maternal responsiveness to the different kinds of infant behaviour, ii) how mothers distributed their responses across the different infant behaviours, using responded infant behaviours as cases, and the type of maternal response as predictor, controlling for the base rate of infant behaviours, iii) the different infant behaviours according to how frequently (% their instances) each kind of maternal behaviour followed, using infant behaviours as cases, response status (e.g., whether mirrored or not mirrored) as dependent variable, and the type of infant behaviour as predictor.

To analyse the effect of maternal responses on the time (seconds) elapsing between consecutive infant social behaviours, we used three-level random intercept Gamma models, with a Log link, with infant age and kind of maternal response (main effects, and interaction) as predictors.

To investigate the role of maternal responsiveness on the development of infant social behaviour over time, we examined the effect of responses at time *t* on the rate of infant social behaviours at time *t* + 1 (i.e., the next assessment), controlling for its rate at time *t*, using two-level random intercept Poisson models, with a Log Link and interaction time (at time *t* + 1) as offset.

For all models, we used Likelihood Ratio Tests (LRT) to assess the effect of the predictors. Marginal *R*^2^ values^48^ are reported for the contribution of predictors to the variance explained by the models. We used Tukey-Kramer contrasts for multiple comparisons. A p-value < 0.05 was considered significant.

## Additional Information

**How to cite this article**: Murray, L. *et al*. The functional architecture of mother-infant communication, and the development of infant social expressiveness in the first two months. *Sci. Rep.* **6**, 39019; doi: 10.1038/srep39019 (2016).

**Publisher's note:** Springer Nature remains neutral with regard to jurisdictional claims in published maps and institutional affiliations.

## Supplementary Material

Supplementary Information
