# Supplementary material for: The functional architecture of mother-infant communication, and the development of infant social expressiveness in the first two months
Source: Sci Rep. 2016 Dec 14;6:39019. doi: 10.1038/srep39019 (PMC5155249; doi:10.1038/srep39019)
Supplement: Supplementary Information [file srep39019-s1.pdf]

SUPPLEMENTARY INFORMATION

The functional architecture of mother-infant communication, and the development of infant social expressiveness in the first two months

Lynne Murray, Leonardo De Pascalis, Laura Bozicevic, Laura Hawkins, Valentina Sclafani, Pier Francesco Ferrari

Supplementary Methods

Participants

Mothers of healthy full-term infants were recruited on the postnatal ward of the Royal Berkshire Hospital, Reading, UK, to a pool of volunteers for child development research at the University of Reading. Twenty mother-infant dyads (12 male, 8 female infants) participated in the study. Infant ages in weeks at the five study assessments were M(SD) 1.47(0.29), 3.09(0.31)), 5.23(0.41), 7.01(0.33)) and 9.14(0.43), and the number of completed assessments per infant was M = 4.55 (SD = 0.61). The sample was generally low risk: mothers were screened to establish they had no mental health problems; their ages ranged from 28.74 to 41.11 years (M = 33.70 (SD= 2.73)), only one was single, and 60% were university graduates. Two thirds (65.00%) were multiparous, and the great majority (90.00%) were White. Infant gestation was M = 40.79 weeks (SD= 1.59), and birthweight was M= 3731.94 gm. (SD = 608.07).

Procedure

Mothers and infants were visited at home at 1, 3, 5, 7 and 9 weeks postpartum by a female researcher who made a three-minute video-recoding of mother-infant face-to-face interaction

on each occasion. To ensure these interactions were as natural as possible, and were conducted when the infant was in an alert state, the mother was asked to feed the infant if appropriate, and then to change their nappy, followed by her being asked to engage with the infant socially as she normally would, without using objects. Filming started when the mother first attempted to engage with the infant. If the infant became distressed and could not be consoled, filming stopped, and was attempted again once the infant had calmed.

Coding

Data for coding were available for 91 of the 100 planned assessments: in 4 instances mothers were not available during the time frame required; in 3 cases there were technical problems with the videos; and in 2 instances infants were distressed throughout. Mother-infant interaction videos were event-coded on a one second time base, using purpose built software for identifying associations between maternal and infant behaviour (details available from LDP). The coding scheme was based on that of Murray and colleagues[1,2] (coding manual available from LM/LB), and included key infant and maternal events described in the literature on early infant social development and mother-infant interactions[3-5]. Coders were graduate students who were blind to study aims and hypotheses, and to background information concerning the infants and their families.

Infant behaviour. Infant behaviours were mutually exclusive, and were clearly discernible, discrete, events with definite onset, and therefore readily identifiable by the mother in live time. Since mothers direct their attention almost exclusively to the infant's face during interactions in the first few months[6], we coded behaviours involving infant facial movements and emissions of sound. Behaviours in group A required the infant to be gazing at their mother's face, as looking at the interactive partner is one of the characteristics of infant communication at this age[6,7], whereas the other groups of infant behaviour were scored independently of the direction of infant gaze.

A. Social behaviours

(i) 'pre-speech'[3]: active movements of lips and tongue (e.g., tongue pushed into the bottom lip, moving it forward, or protruded beyond the lips), and of open mouth shaping (e.g., into an 'O', or pursed, as during cooing, even though unvoiced) that appear to be directed at the mother (Supplementary Figure S1);

(ii) smiles;

(iii) neutral-positive vocalisations, such as cooing.

B. Non-social mouth movements

Mouth movements that appear undirected (e.g., chewing or sucking movements, rolling lips together), or else mouth movements clearly directed to a non-social goal (e.g., rooting to the infant's fist).

C. Negative affect

(i) Mouth -pout, grimace;

(ii) Expression- cry face[8];

(iii) Vocalisation- fuss, cry.

D. Biological events

Sneeze, vomit, posset, hiccough, flatulence, yawn etc.

Maternal behaviours.

1. Contingency: Given that infants of this age cannot detect events as contingent if they occur 3 seconds or more after their own behaviour[9] and that mothers' responses almost always occur within 2 seconds of infant expressions[10], maternal contingent responses were coded as events occurring within two seconds of the infant events above (and they were, therefore, also 'contiguous'), as in[1,2,10].

2. Form: Maternal responses were grouped as follows:

A. Mirroring

Responses of the same valence and intensity[4,11] as the infant's behaviour that are either exact matches, or that match the principal features with some minor modification, i.e., 'enriched' mirroring in which some element is added (such as a vocalisation to a clear mouth opening), 'partial' mirroring in which some element is omitted (such as the facial expression of a cry being imitated but not the sound), or 'modified' mirroring in which the form is slightly changed, often in conventionalised ways (e.g., responding to an infant 'ooo' vocalisation with 'goo') (Supplementary Figure S2).

B. Marking

Responses of the same valence and intensity as the infant's behaviour that single out and 'mark' an infant behaviour with 'attention-attracting' cues[5], without mirroring it.

(i) Positive, with smiles: e.g., infant makes a tongue protrusion and the mother raises head and eyebrows, then nods and clearly smiles, saying 'Is that your tongue?' (Supplementary Figure S3);

(ii) Neutral, without smiles: e.g., infant smiles, and mother responds by raising and lowering her head, and then says 'ooh' with raised brows and an expression of interest (Supplementary Figure S4).

C. Negative responses

i) Rejecting responses: responses that criticize, reject or mock the infant (e.g. the infant's lip is pouted in distress and the mother frowns, shakes her head, and says "oh no, we can't have that", or else responds with a sneer);

ii) Mis-attuned responses - *valence*: Responses where maternal affect is markedly discordant with the infant's behaviour (e.g. the infant shows sign of distress and the mother smiles broadly and laughs);

Mis-attuned responses - *intensity*: a) hyper-responding: responses that are a clear and extreme *exaggeration* in relation to the infant behaviour (e.g., the infant shows minimal mouth opening, and the mother swiftly and strongly moves her head up and down and opens her own mouth very wide); b) hypo-responding: responses that clearly *downplay* the infant behaviour (e.g., the infant makes a strong positive vocalisation, or gives a strong, ‘joyful’ smile, and the mother responds with a flat, dull, vocalization, or minimal, weak smile).

3. Prominence: Maternal non-responsive behaviours were also coded as events; they include spontaneous vocalisations, smiles, tongue protrusions, mouth openings, head nods - behaviours that often occur in attempts to engage with the infant, but that are not contingent on infant events. The number of these behaviours was used to determine the *prominence* of maternal responses, calculated as maternal behaviours that were responses to the infant as a percentage of all maternal behaviours coded (i.e., responses plus non-responsive behaviours).

Coding Inter-rater reliability

Videos were coded by two researchers, who both independently coded the same 20% of the total sample, including one interaction for each mother-infant dyad. Reliability for infant events was as follows: social behaviours $\kappa = .92$; non-social mouth movements $\kappa = .92$; negative affect $\kappa = .85$; biological events $\kappa = .86$. Reliability for maternal responses was as follows: mirroring $\kappa = .90$; positive marking $\kappa = .80$; neutral marking $\kappa = .80$; negative responses $\kappa = .83$. For maternal non-contingent behaviours, the intra-class correlation was 0.87.

Data Analysis

Infant events and maternal responses were each investigated through Principal Components Analysis (PCA), using Parallel Analysis, to determine the number of components to extract,

and Simplimax rotation. We used a generalised linear mixed modelling (GLMM) framework to address the study questions.

Supplementary Results

PCA

Confirming our grouping of infant behaviours, in line with the literature[3,7], three components were extracted that explained 64.33% of the variance (*KMO* = 0.622; Bartlett's Test of Sphericity $X^2_{(28)}$ = 165.055, $P < 0.001$): in the first, behaviours with absolute value loadings >.5 were Pre-speech (.826), Smiles (0.779), and Vocalisations (0.717), that is, positive social behaviour, and, with negative loading, Non-social mouth movements (- 0.572). Behaviours in the second component were Negative Mouth Movements (0.529), Negative Expressions (0.708), and Negative Vocalisations (0.803), that is, infant negative affect; while the third component was Biological Events (0.873).

With regard to maternal responses, two components explained 51.41% of the variance (*KMO* = 0.492; Bartlett's Test of Sphericity $X^2_{(10)}$ = 21.199, p=0.020). Considering absolute value loadings > 0.5, the first included Mirroring (0.826) and Positive Marking (i.e., accompanied by smiles) (0.833). The second component included Rejecting responses (0.670) and Mis-attunements (0.747), that is, the negative responses. Neutral Markings (i.e., without smiles) loaded only weakly on each of these components (0.209 and 0.352, respectively). Although Mirroring and Positive Marking both loaded on the same component, the literature on these responses identifies important distinctions. Thus, Mirroring of the infant's behaviour (usually described in relation to parental mirroring of proprioceptively experienced infant facial movements by visible imitation of the same) has been seen as potentially strengthening[12-15], or forging[16,17] neuronal circuits tuned for decoding social information, and thereby enhancing the infant's own control over the production of the same actions. Marking, by contrast, has been highlighted principally for its referential function[18]. At two-three months, this is often

phatic- i.e., it references the participants' state of engagement, as when the parent greets the infant when eye contact is made[19], but it has also been noted to attribute significance, or meaning, to infant behaviour and expressions[18,20-24,1].Given the theoretical importance of the distinction between mirroring and marking, we retained both the maternal responses comprising the first component as separate variables for data analyses, despite their empirical association (Mirroring and Positive Marking), as well as the Negative responses, and the Neutral Markings.

References

1. Murray, L., Fiori-Cowley, A., Hooper, R. & Cooper, P. The impact of postnatal depression and associated adversity on early mother-infant interactions and later infant outcome. *Child Dev.* **67,** 2512-2526 (1996).

2. Stanley, C., Murray, L. & Stein, A. The effect of postnatal depression on mother-infant interaction, infant response to the still-face perturbation, and performance on an instrumental learning task. *Dev. Psychopathol.* **16,** 1-18 (2004).

3. Trevarthen, C. in *Before Speech: The Beginning of Interpersonal Communication* (ed. Bullowa, M.) 321-348 (Cambridge University Press, 1979).

4. Stern, D. N. *The Interpersonal World of the Infant: A View from Psychoanalysis and Developmental Psychology* (Basic Books, 1985).

5. Csibra, G. & Gergely, G. Natural pedagogy. *Trends Cogn. Sci.* **13,** 148-153 (2009).

6. Murray, L. *et al.* The effect of cleft lip and palate, and the timing of lip repair on

---

[1] A variant of marking, its 'ostensive' form, is more typically used with older infants, and refers to the singling out, at times with the addition of behaviours such as pointing, of some referent for joint attention *other* than the participants' engagement[25,26].

mother-infant interactions and infant development. *J. Child Psychol. Psychiatry* **49,** 115-123 (2008).

7. Lavelli, M. & Fogel, A. Interdyad differences in early mother-infant face-to-face communication: real-time dynamics and developmental pathways. *Dev. Psychol.* **49,** 2257-2271 (2013).

8. Reddy, V., Hay, D., Murray, L. & Trevarthen, C. in *Infant Development: Recent Advances* (eds. Bremner, J. G., Slater, A. & Butterworth, G. E.) 247-273 (Psychology Press, 1997).

9. Gergely, G. & Watson, J. S. in *Early Social Cognition: Understanding Others in the First Months of Life* (ed. Rochat, P.) 101-136 (Lawrence Erlbaum Associates, Inc, 1999).

10. Nicely, P., Tamis-LeMonda, C. S. & Bornstein M. H. (1999) Mothers' attuned responses to infant affect expressivity promote earlier achievement of language milestones. *Infant Behav. Dev.* **22**,557-568.

11. Messinger, D. S. Positive and negative: infant facial expressions and emotions. *Curr. Dir. Psychol. Sci.* **11,** 1-6 (2002).

12. Meltzoff, A. N. 'Like me': a foundation for social cognition. *Dev. Sci.* **10,** 126-134 (2007).

13. Marshall, P. J. & Meltzoff, A. N. Neural mirroring mechanisms and imitation in human infants. *Philos. Trans. R. Soc. Lond. B. Biol. Sci.* **369,** 20130620 (2014).

14. Del Giudice, M. , Manera, V. , Keysers, C. Programmed to learn? The ontogeny of mirror neurons. *Annu. Rev. Neurosci.* **18**, 193-222 (2009)

15. Ferrari, P.F. , Tramacere, A., Simpson, E. A., Iriki, A. Mirror neurons through the lens of epigenetics. *Trends Cogn. Sci*. **17,** 450-457 (2013).

16. Ray, E. & Heyes, C. Imitation in infancy: the wealth of the stimulus. *Dev. Sci.* **14,** 92-105 (2011).

17. Cook, R., Bird, G., Catmur, C., Press, C. & Heyes, C. Mirror neurons: from origin to function. *Behav. Brain Sci.* **37,** 177-192 (2014).

18. Gergely, G. & Watson, J. S. in *Early Social Cognition: Understanding Others in the First Months of Life* (ed. Rochat, P.) 101-136 (Lawrence Erlbaum Associates, Inc, 1999).

19. Snow, C. E. The development of conversation between mothers and babies. *J. Child Lang.* **4**, 1-22 (1977).

20. Kaye, K. Why we don't talk ‘baby talk’to babies. *J. Child Lang*. **7**, 489-507 (1980).

21. Henning, A., Striano, T. & Lieven, E. V. Maternal speech to infants at 1 and 3 months of age. *Infant Behav. Dev*. **28**, 519-536 (2005).

22. Stern, D. N. *The Interpersonal World of the Infant: A View from Psychoanalysis and Developmental Psychology* (Basic Books, 1985).

23. Tronick, E. Z. Why is connection with others so critical? Dyadic meaning making, messiness and complexity governed selective processes which co-create and expand individuals’ states of consciousness. *Emotional development*, 293-315 (2004).

24. Tronick, E. & Beeghly, M. Infants' meaning-making and the development of mental health problems. *American Psychologist* **66**, 107 (2011).

25. O'Neill, M., Bard, K. A., Linnell, M. & Fluck, M. Maternal gestures with 20-month-

old infants in two contexts. *Dev. Sci.* **8**, 352-359 (2005).

26. Gergely, G. Ostensive communication and cultural learning: The natural pedagogy hypothesis. *Agency and joint attention*, 139-151 (2013).

Supplementary Figure S1. Prespeech: Tongue Protrusion and Mouth Opening

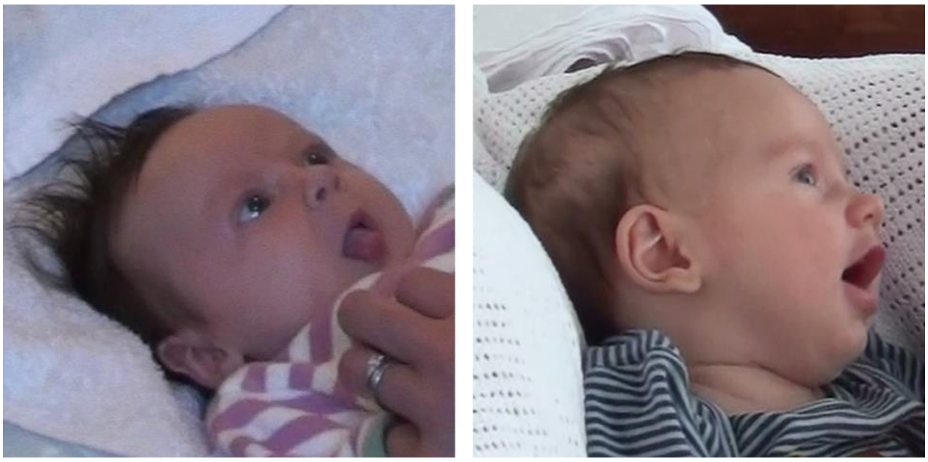

Supplementary Figure S2. Mirroring

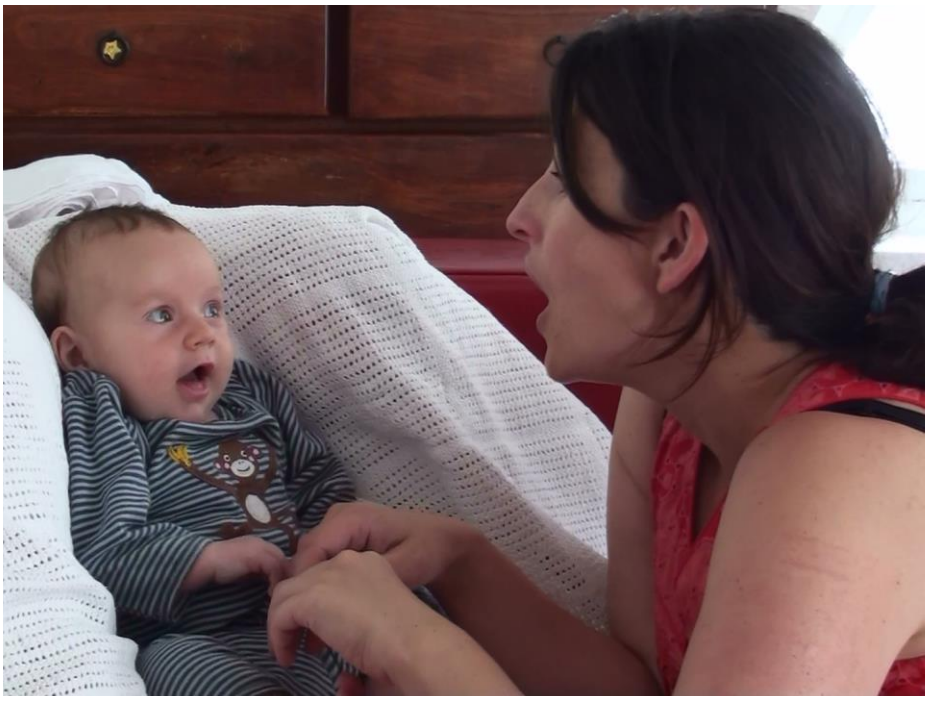

Supplementary Figure S3. Positive marking

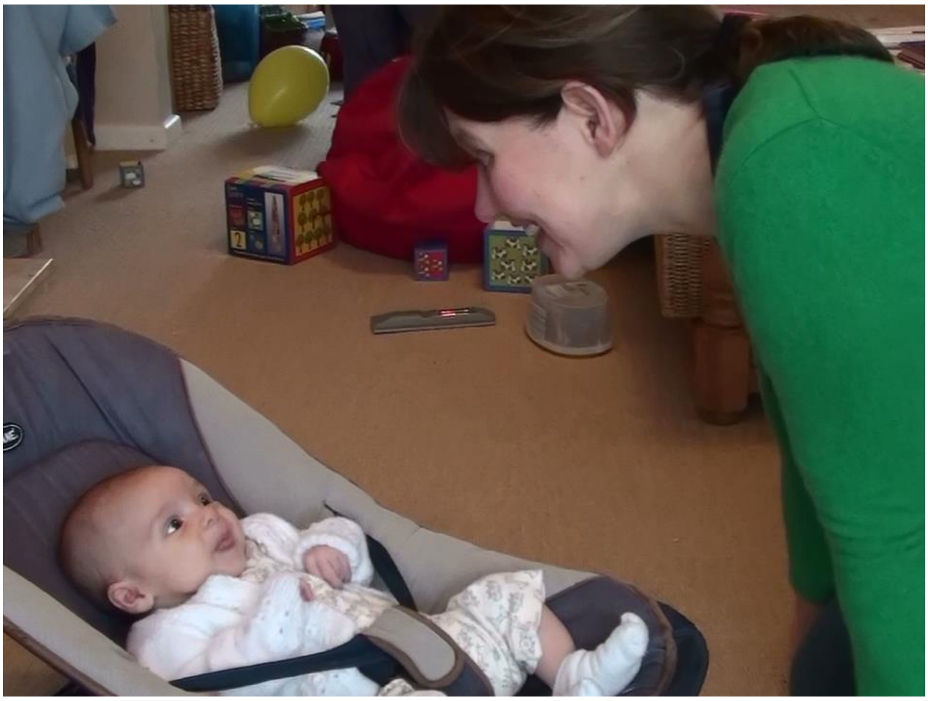

Supplementary Figure S4. Neutral marking

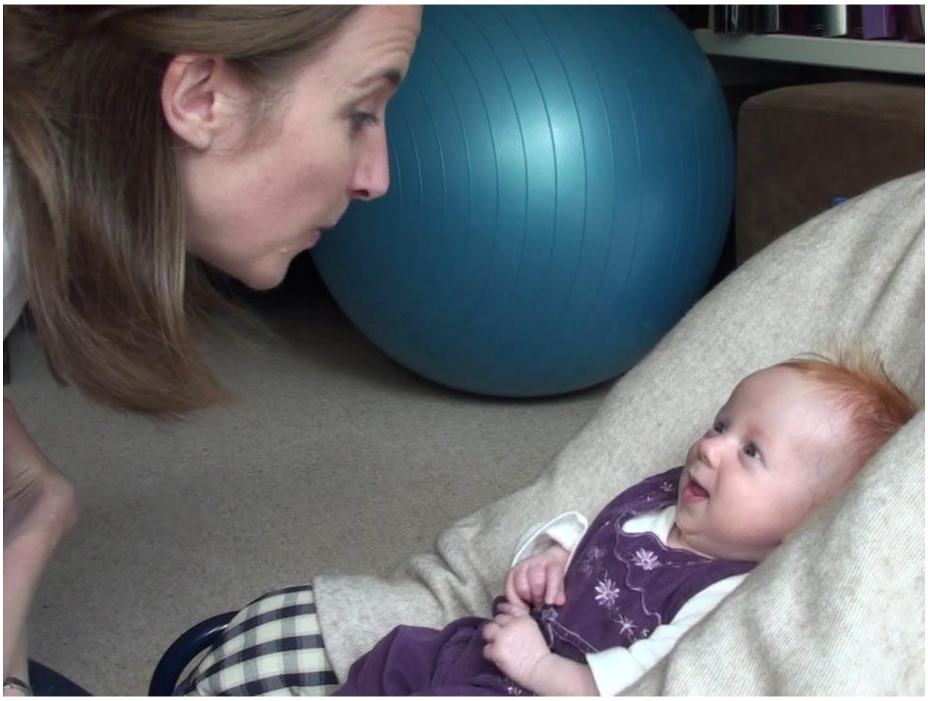

	**Week 1 (N = 18) M (SE)**	**Week 3 (N = 17) M (SE)**	**Week 5 (N = 18) M (SE)**	**Week 7 (N = 19) M (SE)**	**Week 9 (N = 19) M (SE)**
**Biological events**	3.49 (0.55)	5.06 (0.84)	3.71 (0.59)	4.25 (1.00)	2.91 (0.68)
**Negative affect**	1.91 (0.64)	1.40 (0.34)	2.32 (0.77)	0.90 (0.27)	0.40 (0.10)
**Non-social mouth movements**	3.08 (0.55)	3.90 (0.50)	3.08 (0.62)	2.25 (0.42)	0.39 (0.32)
**Social behaviours**	0.44 (0.11)	0.96 (0.25)	2.70 (0.52)	6.23 (1.09)	8.34 (0.84)
Pre-speech	*0.09 (0.06)*	*0.27 (0.10)*	*1.42 (0.35)*	*3.39 (0.81)*	*4.36 (0.56)*
Vocalisations	*0.35 (0.11)*	*0.65 (0.23)*	*1.15 (0.26)*	*2.54 (0.54)*	*3.23 (0.52)*
Smiles	*0.00 (0.00)*	*0.04 (0.03)*	*0.13 (0.05)*	*0.30 (0.10)*	*0.75 (0.18)*

Supplementary Table S1. Means and standard errors for rates per minute of infant behaviours according to infant age.

		Week 1<br>(N = 18)<br>M (SE)	Week 3<br>(N = 17)<br>M (SE)	Week 5<br>(N = 18)<br>M (SE)	Week 7<br>(N = 19)<br>M (SE)	Week 9<br>(N = 19)<br>M (SE)
**Percentages**	**Contingency**	20.81 (4.06)	20.94 (1.88)	26.02 (2.71)	28.11 (2.60)	33.49 (2.97)
	**Prominence**	11.41 (2.11)	9.84 (1.17)	10.63 (1.23)	15.52 (3.27)	15.58 (1.92)
**Rates**	**Mirroring**	0.64 (0.18)	0.31 (0.11)	0.55 (0.18)	1.56 (0.42)	1.87 (0.35)
	**Positive marking**	0.35 (0.22)	0.18 (0.05)	0.56 (0.21)	1.09 (0.25)	1.29 (0.22)
	**Neutral marking**	1.03 (0.21)	1.28 (0.20)	1.05 (0.18)	0.93 (0.16)	0.92 (0.21)
	**Negative responses**	0.52 (0.19)	0.47 (0.18)	0.71 (0.18)	0.37 (0.11)	0.31 (0.08)

Supplementary Table S2. Means and standard errors for percentages of contingency and prominence of maternal behaviour, and for rates per minute of maternal responses, according to infant age.

Infant behaviour	Non-soc. mouth mov. $X^2(3)=20.088$ p<.001				Social behaviour $X^2(3)=32.250$ p<.001				Biological events $X^2(3)=13.070$ p=.004				Negative affect $X^2(3)=50.492$ p<.001			
**Maternal response**	**%**	***Mult. comp.***			**%**	***Mult. comp.***			**%**	***Mult. comp.***			**%**	***Mult. comp.***		
		b	***c***	***d***		***b***	***c***	***d***		***b***	***c***	***d***		***b***	***c***	***d***
a. Mirroring	8.58	*.999*	***.003***	***.028***	60.07	*.999*	***.005***	***<.001***	28.36	*.633*	*.059*	*.983*	2.99	***.039***	***<.001***	***<.001***
b. Positive mark.	8.24	-	***.012***	*.050*	63.74	-	***.009***	***<.001***	22.53	-	***.004***	*.554*	5.49	-	*.594*	***.004***
c. Neutral mark.	4.20	-	-	*.990*	31.68	-	-	*.117*	44.27	-	-	*.332*	19.85	-	-	***.015***
d. Negative resp.	3.39	-	-	-	19.49	-	-	-	30.51	-	-	-	46.61	-	-	-

Supplementary Table S3. Distribution of maternal responses in relation to infant behaviours. Percentages are shown for each of the four different kinds of maternal response as they are distributed across the different kinds of infant behaviour. For each infant behaviour, the p-value for the comparison between maternal responses (controlling for infant age, and the rate per minute of the infant behaviour responded to) is reported. P-values are also shown for multiple comparisons between pairs of maternal responses within each infant behaviour.

Maternal response	Mirroring $X^2(3)=53.681$ $p<.001$				Positive mark. $X^2(3)=28.614$ $p<.001$				Neutral mark. $X^2(3)=103.583$ $p<.001$				Negative resp. $X^2(3)=114.124$ $p<.001$			
**Infant behaviour**	**%**	***Mult. comp.***			**%**	***Mult. comp.***			**%**	***Mult. comp.***			**%**	***Mult. comp.***		
		2	***3***	***4***		***2***	***3***	***4***		***2***	***3***	***4***		***2***	***3***	***4***
1. Non-soc. m. mov.	3.34	***<.001***	***.012***	*.942*	2.18	***<.001***	*.243*	*.502*	1.60	***<.001***	***<.001***	***<.001***	0.58	*.254*	***.001***	***<.001***
2. Social behaviour	15.53	-	***<.001***	***<.001***	11.19	-	***.002***	*.190*	8.00	-	*.591*	*.057*	2.22	-	*.133*	***<.001***
3. Biological events	7.84	-	-	***.045***	4.23	-	-	*.999*	11.96	-	-	*.296*	3.71	-	-	***<.001***
4. Negative affect	2.44	-	-	-	3.05	-	-	-	15.85	-	-	-	16.77	-	-	-

Supplementary Table S4. Infant experience of different maternal responses. Percentages are shown for each of the four different kinds of infant behaviours as they are responded to with the different kinds of maternal response. For each maternal response, the p-value for the comparison between infant behaviours (controlling for infant age) is reported. P-values are also shown for multiple comparisons between pairs of infant behaviours within each maternal response.

Infant Behaviours	Maternal Responses	Stand. Coef.	SE	p	R^2
**All**	**Contingent Responses**	0.095	0.089	.286	.010
**Social**	**Contingent Responses**	0.061	0.120	.611	.006
	**Mirroring**	0.432	0.128	<**.001**	.120
	**Positive Marking**	0.213	0.066	**.001**	.069
	**Neutral Marking**	-0.397	0.155	**.010**	.053
	**Negative Responses**	-0.130	0.052	**.008**	.074
**Non-Social**	**Contingent Responses**	-0.110	0.070	.117	.017
	**Mirroring**	0.099	0.065	.133	.009
	**Positive Marking**	0.197	0.070	**.005**	.051
	**Neutral Marking**	-0.161	0.055	**.003**	.050
	**Negative Responses**	-0.200	0.053	<**.001**	.075

Supplementary Table S5. Standardised coefficients, standard errors, p-values, and R^2 values for the effect of maternal responses at time *t* on the rate of infant social behaviours at time *t+1*.
